# [(4*E*)-3-Ethyl-1-methyl-2,6-di­phenyl­piperidin-4-yl­idene]amino 3-methyl­benzoate

**DOI:** 10.1107/S1600536813024276

**Published:** 2013-09-18

**Authors:** T. Vinuchakkaravarthy, R. Sivakumar, T. Srinivasan, V. Thanikachalam, D. Velmurugan

**Affiliations:** a Centre of Advanced Study in Crystallography and Biophysics, University of Madras, Maraimalai Campus (Guindy Campus), Chennai 600 025, India; b Department of Chemistry, Annamalai University, Annamalai Nagar, Chidambaram 608 002, India

## Abstract

In the title compound, C_28_H_30_N_2_O_2_, the piperidine ring exists in a chair conformation with an equatorial orientation of the phenyl rings and methyl group substituted on the heterocycle. In the crystal, C—H⋯π inter­actions result in chains of mol­ecules running parallel to the *a-*axis direction.

## Related literature

For the synthesis and background to the biological activity of piperidinyl-4-one derivatives, see: Parthiban *et al.* (2009 ▶, 2011 ▶). For crystal structures of related compounds, see: Park *et al.* (2012*a* ▶,*b* ▶). For ring puckering parameters, see: Cremer & Pople (1975 ▶).
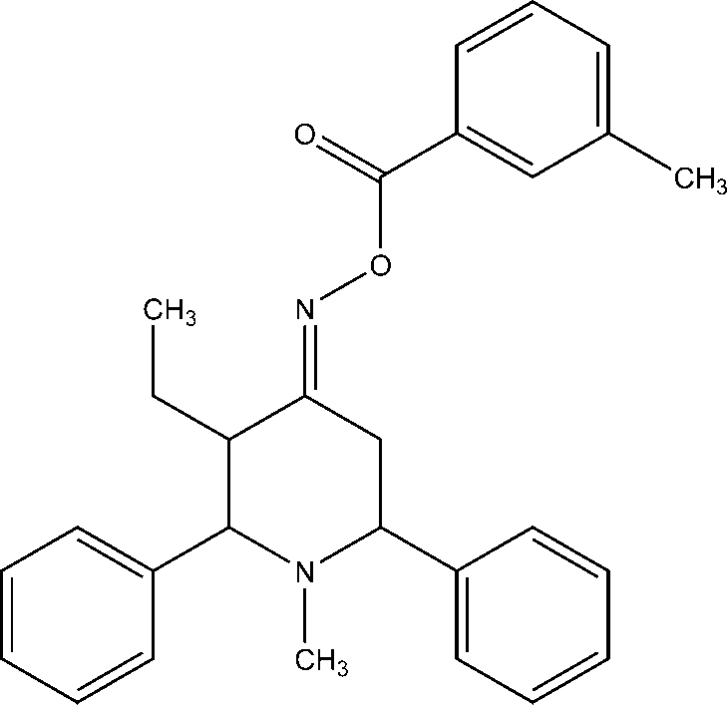


## Experimental

### 

#### Crystal data

C_28_H_30_N_2_O_2_
*M* *_r_* = 426.54
Triclinic, 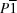

*a* = 10.5220 (15) Å
*b* = 11.8295 (16) Å
*c* = 11.987 (3) Å
α = 112.871 (11)°
β = 97.939 (11)°
γ = 110.123 (8)°
*V* = 1225.5 (4) Å^3^
*Z* = 2
Mo *K*α radiation
μ = 0.07 mm^−1^
*T* = 293 K
0.20 × 0.20 × 0.20 mm

#### Data collection

Bruker SMART APEXII CCD diffractometer
Absorption correction: multi-scan (*SADABS*; Bruker, 2008 ▶) *T*_min_ = 0.986, *T*_max_ = 0.986
18615 measured reflections
5042 independent reflections
3610 reflections with *I* > 2σ(*I*)
*R*_int_ = 0.023

#### Refinement

*R*[*F* ^2^ > 2σ(*F* ^2^)] = 0.046
*wR*(*F* ^2^) = 0.139
*S* = 1.03
5042 reflections
292 parameters
H-atom parameters constrained
Δρ_max_ = 0.15 e Å^−3^
Δρ_min_ = −0.17 e Å^−3^

### 

Data collection: *APEX2* (Bruker, 2008 ▶); cell refinement: *SAINT* (Bruker, 2008 ▶); data reduction: *SAINT*; program(s) used to solve structure: *SHELXS97* (Sheldrick, 2008 ▶); program(s) used to refine structure: *SHELXL97* (Sheldrick, 2008 ▶); molecular graphics: *ORTEP-3 for Windows* (Farrugia, 2012 ▶); software used to prepare material for publication: *SHELXL97* and *PLATON* (Spek, 2009 ▶).

## Supplementary Material

Crystal structure: contains datablock(s) I. DOI: 10.1107/S1600536813024276/pv2635sup1.cif

Structure factors: contains datablock(s) I. DOI: 10.1107/S1600536813024276/pv2635Isup2.hkl

Supplementary material file. DOI: 10.1107/S1600536813024276/pv2635Isup3.cml

Additional supplementary materials:  crystallographic information; 3D view; checkCIF report

## Figures and Tables

**Table 1:** Hydrogen-bond geometry (Å, °) *Cg*1 is the centroid of the C15–C20 ring.

*D*—H⋯*A*	*D*—H	H⋯*A*	*D*⋯*A*	*D*—H⋯*A*
C11—H11⋯*Cg*1^i^	0.93	2.93	3.761 (2)	149
C23—H23⋯*Cg*1^ii^	0.93	2.89	3.730 (3)	151

Symmetry codes: (i) 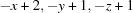; (ii) 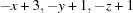.

**Fig. 1.**
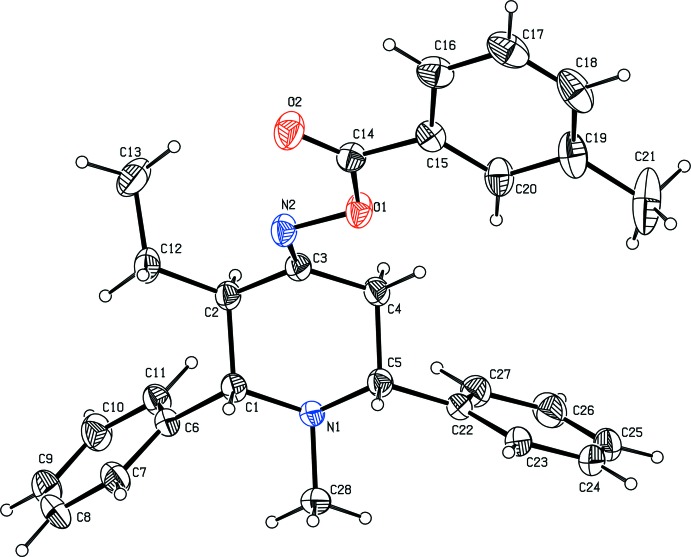
The molecular structure of the title compound with the atom numbering scheme. Displacement ellipsoids are drawn at the 30% probability level. H atoms are presented as small spheres of arbitrary radius.

**Fig. 2.**
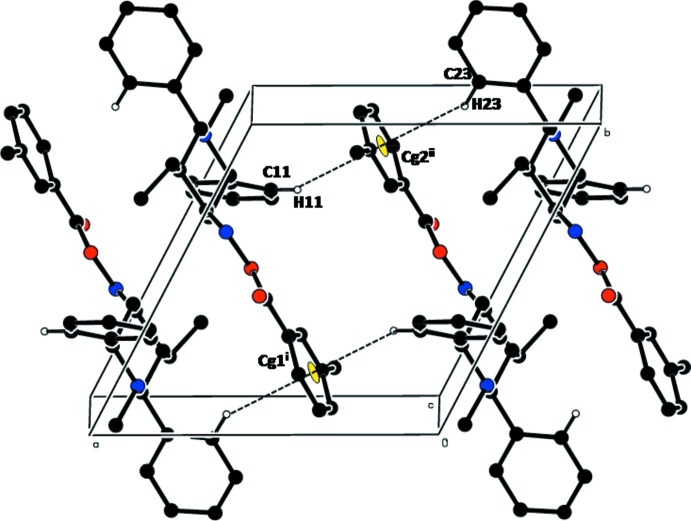
The crystal packing arrangement of the title compound viewed down the b axis showing intermolecular C—H···π hydrogen bond interactions (dashed lines). symmetry codes: (i) 2-X,1-Y,1-Z (ii) 3-X,1-Y,1-Z

## supplementary crystallographic information

### 1. Comment

Piperdin-4-one nucleus is an important pharmacophore due to its broad spectrum of biological actions ranging from antibacterial to anticancer (Parthiban *et al.*, 2009; 2011). Hence, the synthesis and steriochemical analysis of piperdin-4-one nucleus based pharmacophores has gained much interest in the field of medicinal chemistry.

The bond distances and angles in the title compound (Fig. 1) agree very well with the corresponding bond distances and angles reported in closely related compounds (Park *et al.*, 2012*a*; 2012*b*). The piperidone ring (N1/C1—/C5) adopts a chair conformation with puckering parameters: *Q* =0.570 (2) Å, θ = 177.6 (2)° and φ = 3439 (4)° (Cremer & Pople, 1975).

In the crystal, the molecules are stabilized by intermolecular C11—H11···*Cg*1^i^ and C23—H23···*Cg*1^ii^ hydrogen bond interactions, where *Cg*1 is the center of gravity of ring atoms involving C15—C20 of the interacting molecules, respectively (Table 1). The packing of the molecules within the crystal is shown in Fig. 2.

### 2. Experimental

3-Ethyl-2,6-diphenylpiperidin-4-one was synthesized by Mannich condensation using benzaldehyde (2 mol), ammonium acetate (1 mol) and ethyl methyl ketone (1 mol) in absolute ethanol and warmed for 30 min and stirred overnight at room temperature. The product was treated with methyl iodide (1.5 mol) in the presence of potassium carbonate (2 mol) in acetone (10 ml) and refluxed to give 1-methyl-3-ethyl-2,6-diphenylpiperidin-4-one. The oximation was done by hydroxylamine hydrochloride (2 mol) in presence of sodium acetate (2 mol) in ethanol (10 ml) and refluxed. The resulting oxime (0.5 g, 1.55 mmol) was stirred with dry pyridine (5 ml), added 3-methylbenzoic acid (0.23 g, 1.7 mmol) followed by phosphorus oxychloride (0.21 ml, 2.3 mmol) in dropwise addition and stirred at ambient temperature for 15 min; the progress of the reaction was monitored by thin layer chromatography. Upon completion of the reaction, saturated sodium bicarbonate solution (8 ml) was added to the reaction mixture, solid was formed and it was filtered and dried to get a white solid (0.58 g, 87.8%) which was recrystallized from ethanol to yield crystals suitable for X-ray crystallographic studies.

### 3. Refinement

H atoms were positioned geometrically (C—H = 0.93–0.98 Å) and allowed to ride on their parent atoms, with *U*_iso_(H) = 1.5*U*_eq_(C) for methyl H and 1.2*U*_eq_(C) for other H atoms.

### Crystal data

**Table:**

C_28_H_30_N_2_O_2_	*Z* = 2
*M**_r_* = 426.54	*F*(000) = 456
Triclinic, *P*1	*D*_x_ = 1.156 Mg m^−^^3^
Hall symbol: -P 1	Mo *K*α radiation, λ = 0.71073 Å
*a* = 10.5220 (15) Å	Cell parameters from 5087 reflections
*b* = 11.8295 (16) Å	θ = 1.9–26.5°
*c* = 11.987 (3) Å	µ = 0.07 mm^−^^1^
α = 112.871 (11)°	*T* = 293 K
β = 97.939 (11)°	Block, colorless
γ = 110.123 (8)°	0.20 × 0.20 × 0.20 mm
*V* = 1225.5 (4) Å^3^	

### Data collection

**Table:**

Bruker SMART APEXII CCD diffractometer	5042 independent reflections
Radiation source: fine-focus sealed tube	3610 reflections with *I* > 2σ(*I*)
Graphite monochromator	*R*_int_ = 0.023
ω and φ scans	θ_max_ = 26.5°, θ_min_ = 1.9°
Absorption correction: multi-scan (*SADABS*; Bruker, 2008)	*h* = −13→13
*T*_min_ = 0.986, *T*_max_ = 0.986	*k* = −14→14
18615 measured reflections	*l* = −15→15

### Refinement

**Table:**

Refinement on *F*^2^	Primary atom site location: structure-invariant direct methods
Least-squares matrix: full	Secondary atom site location: difference Fourier map
*R*[*F*^2^ > 2σ(*F*^2^)] = 0.046	Hydrogen site location: inferred from neighbouring sites
*wR*(*F*^2^) = 0.139	H-atom parameters constrained
*S* = 1.03	*w* = 1/[σ^2^(*F*_o_^2^) + (0.0639*P*)^2^ + 0.1881*P*] where *P* = (*F*_o_^2^ + 2*F*_c_^2^)/3
5042 reflections	(Δ/σ)_max_ < 0.001
292 parameters	Δρ_max_ = 0.15 e Å^−^^3^
0 restraints	Δρ_min_ = −0.17 e Å^−^^3^

### Special details

**Table:**

Geometry. All e.s.d.'s (except the e.s.d. in the dihedral angle between two l.s. planes) are estimated using the full covariance matrix. The cell e.s.d.'s are taken into account individually in the estimation of e.s.d.'s in distances, angles and torsion angles; correlations between e.s.d.'s in cell parameters are only used when they are defined by crystal symmetry. An approximate (isotropic) treatment of cell e.s.d.'s is used for estimating e.s.d.'s involving l.s. planes.
Refinement. Refinement of *F*^2^ against ALL reflections. The weighted *R*-factor *wR* and goodness of fit *S* are based on *F*^2^, conventional *R*-factors *R* are based on *F*, with *F* set to zero for negative *F*^2^. The threshold expression of *F*^2^ > σ(*F*^2^) is used only for calculating *R*-factors(gt) *etc*. and is not relevant to the choice of reflections for refinement. *R*-factors based on *F*^2^ are statistically about twice as large as those based on *F*, and *R*- factors based on ALL data will be even larger.

### Fractional atomic coordinates and isotropic or equivalent isotropic displacement parameters (Å^2^)

**Table:**

	*x*	*y*	*z*	*U*_iso_*/*U*_eq_	
C1	0.89609 (15)	0.09484 (14)	0.34733 (14)	0.0517 (3)	
H1	0.9758	0.0793	0.3798	0.062*	
C2	0.88999 (15)	0.21364 (15)	0.45884 (13)	0.0532 (3)	
H2	0.8121	0.2300	0.4241	0.064*	
C3	1.02600 (15)	0.33834 (14)	0.49960 (14)	0.0535 (3)	
C4	1.04934 (17)	0.37330 (15)	0.39518 (14)	0.0571 (4)	
H4A	1.1390	0.4536	0.4267	0.069*	
H4B	0.9733	0.3934	0.3654	0.069*	
C5	1.05203 (15)	0.25298 (14)	0.28511 (13)	0.0517 (3)	
H5	1.1339	0.2390	0.3154	0.062*	
C6	0.76059 (16)	−0.03556 (14)	0.29564 (14)	0.0542 (4)	
C7	0.7643 (2)	−0.15236 (18)	0.29179 (19)	0.0775 (5)	
H7	0.8509	−0.1509	0.3238	0.093*	
C8	0.6392 (3)	−0.27266 (19)	0.2404 (2)	0.0977 (7)	
H8	0.6432	−0.3515	0.2361	0.117*	
C9	0.5122 (3)	−0.2753 (2)	0.1966 (2)	0.0937 (6)	
H9	0.4288	−0.3555	0.1635	0.112*	
C10	0.5065 (2)	−0.1611 (2)	0.20100 (19)	0.0807 (5)	
H10	0.4189	−0.1630	0.1713	0.097*	
C11	0.62958 (17)	−0.04179 (16)	0.24912 (16)	0.0639 (4)	
H11	0.6241	0.0354	0.2502	0.077*	
C12	0.85682 (19)	0.18214 (18)	0.56631 (16)	0.0724 (5)	
H12A	0.7807	0.0907	0.5294	0.087*	
H12B	0.9405	0.1839	0.6139	0.087*	
C13	0.8129 (2)	0.2812 (3)	0.6574 (2)	0.1013 (7)	
H13A	0.8910	0.3707	0.7004	0.152*	
H13B	0.7872	0.2525	0.7191	0.152*	
H13C	0.7327	0.2832	0.6105	0.152*	
C14	1.31474 (16)	0.59266 (16)	0.75477 (14)	0.0573 (4)	
C15	1.43652 (16)	0.71158 (16)	0.76655 (15)	0.0590 (4)	
C16	1.51466 (19)	0.81891 (18)	0.88722 (18)	0.0763 (5)	
H16	1.4912	0.8145	0.9579	0.092*	
C17	1.6272 (2)	0.9324 (2)	0.9027 (3)	0.0959 (7)	
H17	1.6793	1.0050	0.9839	0.115*	
C18	1.6625 (2)	0.9387 (2)	0.7998 (3)	0.0988 (8)	
H18	1.7391	1.0160	0.8119	0.119*	
C19	1.5872 (2)	0.8331 (2)	0.6777 (2)	0.0920 (7)	
C20	1.47272 (18)	0.71950 (19)	0.66290 (18)	0.0723 (5)	
H20	1.4196	0.6475	0.5815	0.087*	
C21	1.6253 (3)	0.8389 (4)	0.5631 (3)	0.1622 (15)	
H21A	1.5714	0.8753	0.5285	0.243*	
H21B	1.6037	0.7487	0.4997	0.243*	
H21C	1.7253	0.8963	0.5878	0.243*	
C22	1.07151 (17)	0.28834 (14)	0.17812 (14)	0.0547 (4)	
C23	1.19935 (19)	0.31668 (16)	0.15166 (16)	0.0679 (4)	
H23	1.2729	0.3102	0.1977	0.081*	
C24	1.2191 (3)	0.35446 (18)	0.0577 (2)	0.0875 (6)	
H24	1.3055	0.3727	0.0408	0.105*	
C25	1.1128 (3)	0.36531 (19)	−0.0108 (2)	0.0965 (7)	
H25	1.1268	0.3912	−0.0738	0.116*	
C26	0.9851 (3)	0.3376 (2)	0.01431 (18)	0.0893 (6)	
H26	0.9123	0.3446	−0.0321	0.107*	
C27	0.9640 (2)	0.29925 (17)	0.10809 (15)	0.0687 (4)	
H27	0.8771	0.2807	0.1243	0.082*	
C28	0.9312 (2)	0.01436 (16)	0.14152 (17)	0.0701 (5)	
H28A	0.9489	0.0364	0.0744	0.105*	
H28B	0.8433	−0.0664	0.1083	0.105*	
H28C	1.0079	−0.0012	0.1756	0.105*	
N1	0.92147 (12)	0.12787 (11)	0.24286 (11)	0.0493 (3)	
N2	1.10892 (13)	0.39464 (13)	0.61306 (12)	0.0616 (3)	
O1	1.23463 (11)	0.50961 (11)	0.63113 (10)	0.0681 (3)	
O2	1.28828 (14)	0.57319 (14)	0.84073 (11)	0.0882 (4)	

### Atomic displacement parameters (Å^2^)

**Table:**

	*U*^11^	*U*^22^	*U*^33^	*U*^12^	*U*^13^	*U*^23^
C1	0.0478 (8)	0.0499 (8)	0.0622 (8)	0.0209 (7)	0.0187 (7)	0.0307 (7)
C2	0.0504 (8)	0.0505 (8)	0.0542 (8)	0.0156 (7)	0.0179 (6)	0.0255 (7)
C3	0.0539 (8)	0.0482 (8)	0.0518 (8)	0.0164 (7)	0.0176 (7)	0.0222 (7)
C4	0.0621 (9)	0.0454 (8)	0.0526 (8)	0.0131 (7)	0.0163 (7)	0.0221 (7)
C5	0.0467 (8)	0.0494 (8)	0.0586 (8)	0.0176 (7)	0.0188 (6)	0.0270 (7)
C6	0.0576 (9)	0.0454 (8)	0.0618 (8)	0.0195 (7)	0.0245 (7)	0.0279 (7)
C7	0.0865 (13)	0.0599 (11)	0.1044 (14)	0.0362 (10)	0.0408 (11)	0.0483 (10)
C8	0.126 (2)	0.0494 (11)	0.1286 (18)	0.0337 (12)	0.0604 (16)	0.0492 (12)
C9	0.0915 (16)	0.0567 (12)	0.1021 (15)	0.0041 (11)	0.0354 (12)	0.0313 (11)
C10	0.0603 (11)	0.0702 (12)	0.0894 (13)	0.0080 (9)	0.0193 (9)	0.0360 (10)
C11	0.0579 (9)	0.0524 (9)	0.0750 (10)	0.0160 (8)	0.0192 (8)	0.0312 (8)
C12	0.0696 (11)	0.0687 (11)	0.0636 (10)	0.0087 (9)	0.0225 (8)	0.0348 (9)
C13	0.0995 (16)	0.1203 (18)	0.0744 (12)	0.0333 (14)	0.0482 (11)	0.0423 (12)
C14	0.0551 (9)	0.0585 (9)	0.0520 (8)	0.0213 (8)	0.0133 (7)	0.0241 (7)
C15	0.0485 (8)	0.0579 (9)	0.0647 (9)	0.0204 (7)	0.0097 (7)	0.0285 (8)
C16	0.0624 (11)	0.0660 (11)	0.0757 (11)	0.0228 (9)	0.0083 (9)	0.0190 (9)
C17	0.0648 (12)	0.0629 (12)	0.1178 (18)	0.0174 (10)	0.0008 (12)	0.0214 (12)
C18	0.0517 (11)	0.0727 (14)	0.159 (2)	0.0133 (10)	0.0089 (13)	0.0625 (16)
C19	0.0600 (11)	0.1018 (16)	0.1250 (18)	0.0199 (12)	0.0197 (12)	0.0798 (15)
C20	0.0551 (9)	0.0785 (12)	0.0759 (11)	0.0147 (9)	0.0111 (8)	0.0449 (10)
C21	0.1018 (19)	0.216 (4)	0.180 (3)	0.011 (2)	0.0387 (19)	0.156 (3)
C22	0.0625 (9)	0.0416 (7)	0.0548 (8)	0.0174 (7)	0.0242 (7)	0.0205 (6)
C23	0.0717 (11)	0.0522 (9)	0.0771 (11)	0.0199 (8)	0.0379 (9)	0.0292 (8)
C24	0.1104 (16)	0.0569 (11)	0.0910 (14)	0.0218 (11)	0.0622 (13)	0.0335 (10)
C25	0.157 (2)	0.0591 (11)	0.0719 (12)	0.0315 (13)	0.0545 (14)	0.0364 (10)
C26	0.1257 (18)	0.0718 (12)	0.0653 (11)	0.0371 (12)	0.0196 (11)	0.0359 (10)
C27	0.0769 (11)	0.0651 (10)	0.0615 (9)	0.0275 (9)	0.0206 (8)	0.0307 (8)
C28	0.0791 (11)	0.0522 (9)	0.0778 (11)	0.0282 (9)	0.0415 (9)	0.0241 (8)
N1	0.0489 (7)	0.0413 (6)	0.0549 (6)	0.0166 (5)	0.0219 (5)	0.0207 (5)
N2	0.0531 (7)	0.0586 (8)	0.0584 (8)	0.0078 (6)	0.0162 (6)	0.0285 (6)
O1	0.0575 (7)	0.0676 (7)	0.0544 (6)	0.0030 (6)	0.0117 (5)	0.0287 (5)
O2	0.0914 (9)	0.0885 (9)	0.0587 (7)	0.0120 (7)	0.0212 (6)	0.0347 (7)

### Geometric parameters (Å, º)

**Table:**

C1—N1	1.4809 (17)	C14—O1	1.3511 (18)
C1—C6	1.515 (2)	C14—C15	1.482 (2)
C1—C2	1.547 (2)	C15—C20	1.376 (2)
C1—H1	0.9800	C15—C16	1.381 (2)
C2—C3	1.502 (2)	C16—C17	1.375 (3)
C2—C12	1.526 (2)	C16—H16	0.9300
C2—H2	0.9800	C17—C18	1.358 (3)
C3—N2	1.2740 (19)	C17—H17	0.9300
C3—C4	1.4884 (19)	C18—C19	1.381 (3)
C4—C5	1.532 (2)	C18—H18	0.9300
C4—H4A	0.9700	C19—C20	1.390 (2)
C4—H4B	0.9700	C19—C21	1.503 (3)
C5—N1	1.4699 (17)	C20—H20	0.9300
C5—C22	1.5142 (19)	C21—H21A	0.9600
C5—H5	0.9800	C21—H21B	0.9600
C6—C11	1.379 (2)	C21—H21C	0.9600
C6—C7	1.379 (2)	C22—C23	1.383 (2)
C7—C8	1.394 (3)	C22—C27	1.387 (2)
C7—H7	0.9300	C23—C24	1.380 (3)
C8—C9	1.352 (3)	C23—H23	0.9300
C8—H8	0.9300	C24—C25	1.367 (3)
C9—C10	1.354 (3)	C24—H24	0.9300
C9—H9	0.9300	C25—C26	1.375 (3)
C10—C11	1.380 (2)	C25—H25	0.9300
C10—H10	0.9300	C26—C27	1.384 (2)
C11—H11	0.9300	C26—H26	0.9300
C12—C13	1.516 (3)	C27—H27	0.9300
C12—H12A	0.9700	C28—N1	1.4666 (19)
C12—H12B	0.9700	C28—H28A	0.9600
C13—H13A	0.9600	C28—H28B	0.9600
C13—H13B	0.9600	C28—H28C	0.9600
C13—H13C	0.9600	N2—O1	1.4492 (16)
C14—O2	1.1883 (18)		
			
N1—C1—C6	109.65 (11)	O2—C14—O1	124.24 (15)
N1—C1—C2	111.57 (11)	O2—C14—C15	125.66 (14)
C6—C1—C2	111.31 (11)	O1—C14—C15	110.08 (13)
N1—C1—H1	108.1	C20—C15—C16	119.39 (16)
C6—C1—H1	108.1	C20—C15—C14	122.82 (15)
C2—C1—H1	108.1	C16—C15—C14	117.79 (16)
C3—C2—C12	114.71 (12)	C17—C16—C15	119.9 (2)
C3—C2—C1	107.06 (11)	C17—C16—H16	120.1
C12—C2—C1	113.67 (12)	C15—C16—H16	120.1
C3—C2—H2	107.0	C18—C17—C16	120.2 (2)
C12—C2—H2	107.0	C18—C17—H17	119.9
C1—C2—H2	107.0	C16—C17—H17	119.9
N2—C3—C4	128.60 (13)	C17—C18—C19	121.6 (2)
N2—C3—C2	117.74 (13)	C17—C18—H18	119.2
C4—C3—C2	113.50 (12)	C19—C18—H18	119.2
C3—C4—C5	109.35 (12)	C18—C19—C20	117.8 (2)
C3—C4—H4A	109.8	C18—C19—C21	122.1 (2)
C5—C4—H4A	109.8	C20—C19—C21	120.2 (2)
C3—C4—H4B	109.8	C15—C20—C19	121.16 (18)
C5—C4—H4B	109.8	C15—C20—H20	119.4
H4A—C4—H4B	108.3	C19—C20—H20	119.4
N1—C5—C22	111.99 (11)	C19—C21—H21A	109.5
N1—C5—C4	110.54 (11)	C19—C21—H21B	109.5
C22—C5—C4	108.78 (11)	H21A—C21—H21B	109.5
N1—C5—H5	108.5	C19—C21—H21C	109.5
C22—C5—H5	108.5	H21A—C21—H21C	109.5
C4—C5—H5	108.5	H21B—C21—H21C	109.5
C11—C6—C7	117.80 (15)	C23—C22—C27	118.32 (15)
C11—C6—C1	121.02 (12)	C23—C22—C5	120.77 (15)
C7—C6—C1	121.17 (14)	C27—C22—C5	120.84 (14)
C6—C7—C8	120.51 (19)	C24—C23—C22	120.75 (19)
C6—C7—H7	119.7	C24—C23—H23	119.6
C8—C7—H7	119.7	C22—C23—H23	119.6
C9—C8—C7	120.33 (18)	C25—C24—C23	120.65 (19)
C9—C8—H8	119.8	C25—C24—H24	119.7
C7—C8—H8	119.8	C23—C24—H24	119.7
C8—C9—C10	119.89 (19)	C24—C25—C26	119.32 (18)
C8—C9—H9	120.1	C24—C25—H25	120.3
C10—C9—H9	120.1	C26—C25—H25	120.3
C9—C10—C11	120.6 (2)	C25—C26—C27	120.5 (2)
C9—C10—H10	119.7	C25—C26—H26	119.7
C11—C10—H10	119.7	C27—C26—H26	119.7
C6—C11—C10	120.88 (16)	C26—C27—C22	120.43 (18)
C6—C11—H11	119.6	C26—C27—H27	119.8
C10—C11—H11	119.6	C22—C27—H27	119.8
C13—C12—C2	113.43 (16)	N1—C28—H28A	109.5
C13—C12—H12A	108.9	N1—C28—H28B	109.5
C2—C12—H12A	108.9	H28A—C28—H28B	109.5
C13—C12—H12B	108.9	N1—C28—H28C	109.5
C2—C12—H12B	108.9	H28A—C28—H28C	109.5
H12A—C12—H12B	107.7	H28B—C28—H28C	109.5
C12—C13—H13A	109.5	C28—N1—C5	108.74 (11)
C12—C13—H13B	109.5	C28—N1—C1	109.56 (11)
H13A—C13—H13B	109.5	C5—N1—C1	113.06 (11)
C12—C13—H13C	109.5	C3—N2—O1	108.75 (11)
H13A—C13—H13C	109.5	C14—O1—N2	113.11 (11)
H13B—C13—H13C	109.5		
			
N1—C1—C2—C3	−55.08 (15)	C16—C17—C18—C19	0.3 (3)
C6—C1—C2—C3	−177.93 (11)	C17—C18—C19—C20	0.4 (3)
N1—C1—C2—C12	177.19 (12)	C17—C18—C19—C21	−179.9 (2)
C6—C1—C2—C12	54.34 (16)	C16—C15—C20—C19	0.5 (3)
C12—C2—C3—N2	9.1 (2)	C14—C15—C20—C19	179.61 (16)
C1—C2—C3—N2	−118.05 (15)	C18—C19—C20—C15	−0.8 (3)
C12—C2—C3—C4	−175.05 (13)	C21—C19—C20—C15	179.5 (2)
C1—C2—C3—C4	57.83 (16)	N1—C5—C22—C23	−129.64 (14)
N2—C3—C4—C5	116.68 (18)	C4—C5—C22—C23	107.88 (16)
C2—C3—C4—C5	−58.66 (17)	N1—C5—C22—C27	53.35 (18)
C3—C4—C5—N1	55.01 (16)	C4—C5—C22—C27	−69.13 (17)
C3—C4—C5—C22	178.37 (12)	C27—C22—C23—C24	−0.2 (2)
N1—C1—C6—C11	−65.70 (17)	C5—C22—C23—C24	−177.26 (14)
C2—C1—C6—C11	58.24 (18)	C22—C23—C24—C25	0.3 (3)
N1—C1—C6—C7	113.34 (16)	C23—C24—C25—C26	−0.3 (3)
C2—C1—C6—C7	−122.72 (16)	C24—C25—C26—C27	0.1 (3)
C11—C6—C7—C8	1.1 (3)	C25—C26—C27—C22	0.0 (3)
C1—C6—C7—C8	−178.01 (16)	C23—C22—C27—C26	0.0 (2)
C6—C7—C8—C9	−1.7 (3)	C5—C22—C27—C26	177.10 (15)
C7—C8—C9—C10	0.9 (3)	C22—C5—N1—C28	61.00 (15)
C8—C9—C10—C11	0.4 (3)	C4—C5—N1—C28	−177.53 (12)
C7—C6—C11—C10	0.3 (2)	C22—C5—N1—C1	−177.08 (11)
C1—C6—C11—C10	179.37 (15)	C4—C5—N1—C1	−55.61 (14)
C9—C10—C11—C6	−1.1 (3)	C6—C1—N1—C28	−58.12 (15)
C3—C2—C12—C13	71.5 (2)	C2—C1—N1—C28	178.09 (12)
C1—C2—C12—C13	−164.88 (15)	C6—C1—N1—C5	−179.58 (11)
O2—C14—C15—C20	170.94 (17)	C2—C1—N1—C5	56.63 (15)
O1—C14—C15—C20	−10.5 (2)	C4—C3—N2—O1	3.1 (2)
O2—C14—C15—C16	−10.0 (2)	C2—C3—N2—O1	178.31 (12)
O1—C14—C15—C16	168.57 (14)	O2—C14—O1—N2	1.7 (2)
C20—C15—C16—C17	0.2 (3)	C15—C14—O1—N2	−176.83 (11)
C14—C15—C16—C17	−178.97 (16)	C3—N2—O1—C14	166.95 (14)
C15—C16—C17—C18	−0.6 (3)		

### Hydrogen-bond geometry (Å, º)

Cg1 is the centroid of the C15–C20 ring.

**Table:**

*D*—H···*A*	*D*—H	H···*A*	*D*···*A*	*D*—H···*A*
C11—H11···*Cg*1^i^	0.93	2.93	3.761 (2)	149
C23—H23···*Cg*1^ii^	0.93	2.89	3.730 (3)	151

Symmetry codes: (i) −*x*+2, −*y*+1, −*z*+1; (ii) −*x*+3, −*y*+1, −*z*+1.
